# Effects of *Stathmin 1* Gene Knockout on Behaviors and Dopaminergic Markers in Mice Exposed to Social Defeat Stress

**DOI:** 10.3390/brainsci9090215

**Published:** 2019-08-26

**Authors:** Thong Ba Nguyen, Vishwanath Vasudev Prabhu, Yan Hong Piao, Young Eun Oh, Rami Fatima Zahra, Young-Chul Chung

**Affiliations:** 1 Department of Psychiatry, Chonbuk National University Medical School, Jeonju 561-712, Korea; 2 Research Institute of Clinical Medicine of Chonbuk National University, Biomedical Research Institute of Chonbuk National University Hospital, Jeonju 561-712, Korea; 3 Department of Physical Therapy, East Carolina University, Greenville, NC 27834, USA

**Keywords:** Stathmin 1, social defeat stress, novelty object recognition test, social interaction, D2 receptor isoforms, DARPP-32

## Abstract

Stathmin (*STMN*), a microtubule-destabilizing factor, can regulate fear, anxiety, and learning. Social defeat stress (SDS) has detrimental effects on mental health and increases the risk of various psychiatric diseases. This study investigated the effects of *STMN1* gene knockout (KO) on behavioral parameters and dopaminergic markers using an SDS mouse model. The *STMN1* KO mice showed anxious hyperactivity, impaired object recognition, and decreased levels of neutral and social investigating behaviors at baseline compared to wild-type (WT) mice. The impact of SDS on neutral, social investigating and dominant behaviors differed markedly between the *STMN1* WT and KO mice. In addition, different levels of total DARPP-32 and pDARPP-32 Thr75 expression were observed among the control, unsusceptible, and susceptible groups of *STMN1* KO mice. Our results show that *STMN1* has specific roles in locomotion, object recognition, and social interactions. Moreover, SDS had differential impacts on social interactions and dopaminergic markers between *STMN1* WT and KO mice.

## 1. Introduction

The stathmin (*STMN*) tubulin-binding proteins, including *STMN1*, *SCG10 (STMN2)*, *SCLIP (STMN3)*, and *RB3 (STMN4)*, are highly expressed during early postnatal brain development [1,2]. In particular, *STMN1* is involved in the formation and disassembly of microtubules (MTs) [1,3] and plays an important role in neurite outgrowth and synaptic plasticity [4]. *STMN1* is a cytosolic phosphoprotein [5] that interacts with tubulin heterodimers and prevents them from forming MTs [6]. After phosphorylation, *STMN* releases tubulin, allowing MT formation. In adult rodents, the highest levels of *STMN1* expression are found in the prefrontal cortex (PFC) and nucleus accumbens [7], as well as in the lateral nucleus of the amygdala (AMY) and related thalamo-cortical structures [8]. Immunostaining showed that *STMN1* was present in many of the same locations in adult humans and rodents [9]. *STMN1* was shown to be involved in processing fear in both mice [8] and humans [10]. Significant correlations between the *STMN1* gene and a broad range of neuropsychiatric disorders that involve dysfunctional neuronal networking have been reported, including neurodegenerative disorders and schizophrenia [11,12], autism spectrum disorders, anxiety disorders [13], depression and attention-deficit/hyperactivity disorder [14], as well as post-traumatic disorder [15,16]. 

*STMN* knockout (KO) mice (*STMN*^−/−^) show increased MT stability in the AMY. This leads to deficiencies in long-term potentiation (LTP) in the AMY but does not affect basal synaptic transmission or dendritic morphology [8]. The corresponding behavioral phenotype includes deficiencies in innate and learned responses to fear [8], as well as an enhanced fear extinction response [17], but normal pain sensitivity and hippocampus (HIP)-dependent spatial memory in a water maze [8]. Therefore, changes in *STMN*^−/−^ mice appear to be specific to processing responses to fear in the AMY. Consequently, *STMN*^−/−^ mice are a good animal model for studying the role of the AMY in behaviors that depend on threat or fear assessment.

Social defeat stress (SDS) is a type of social stress induced by exposure to a dominant conspecific subject. Defeat can induce a wide range of emotional (e.g., fear, anxiety, and depression) and behavioral abnormalities [18]. The SDS paradigm has been used widely as an animal model for depression, anxiety disorders [19], and possibly schizophrenia [20,21]. Dopamine (DA) plays a pivotal role in regulating threat-related emotional memory and also modulates cognitive functions, including reward, motivation, and salience [22,23]. We considered the SDS model ideal for studying the effects of knocking out the fear gene *STMN1* on dopaminergic markers involved in processing responses to fear. This study investigated the effects of knocking out the *STMN1* gene on behavioral parameters and dopaminergic markers using the SDS model in mice.

## 2. Materials and Methods

### 2.1. Experimental Animal

Experiments were conducted using male homozygous *STMN1* KO and wild-type (WT) mice born from heterozygous mutants (*STMN1*^+/−^) maintained on C57BL/6J background (purchased from the Jackson laboratory (strain name: B6.129P2-*STMN1*^tm1Wed^/J; stock number: 012915). After genotyping, mice were housed (*n* = 4–5 mice per cage) in a fully climate-controlled room at constant temperature 22 ± 1 °C, humidity on a normal 12h light/dark cycle (lights turn on 8 a.m. to 8 p.m.), and food and water available ad libitum. All experiments were made in a strict accordance with the recommendations in the Guidelines for animals from institutional animal care and use committee of Chonbuk national university (IACUC) and the National Institutes of Health (NIH) principles for the Care and Use of Laboratory Animals based on the 3Rs (replacement, refinement and reduction) and compliance with the Animal Care Committee of Chonbuk national medical school (Approval number: CBU 2018-00213).

### 2.2. Study Design

**Figure 1:**
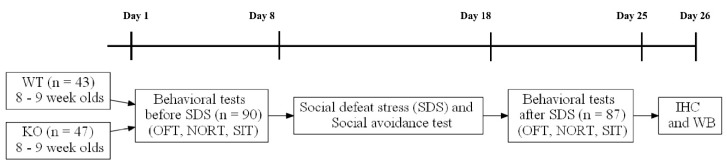
Experimental procedure.

Mice aged about 8–9 weeks and weighing 20–23 g were exposed to behavioral tests in order of stress intensity (i.e., open field test, novel object recognition test, and social interaction test) before social defeat stress for one week. After SDS for 10 days, social avoidance tests were performed at day 18. Then, same behavioral tests were performed again for one week. After 24 h from the last behavioral test, the mice were killed by cervical dislocation and brain tissues were obtained for molecular studies (Figure 1).

### 2.3. Behavioral Measures

The following tests were performed before and after SDS: novel object recognition test (NORT), open field test (OFT) and social interaction test (SIT). The detailed procedures were described in our previous studies [24,25,26].

#### 2.3.1. Social Defeat Stress (SDS) 

Male CD-1 retired breeder mice (Central Lab Inc., Japan) were screened for aggressiveness based on two criteria: during three 180 s screening sessions, once daily, the CD-1 mouse must attack in at least two consecutive sessions; and the latency to initial aggression, which was recorded during each session, must be less than 60 s [27]. C57BL/6J mice were introduced into the home cage of an unfamiliar CD1 aggressor mouse and they were allowed to interact for 5 min. We intervened to stop serious or prolonged confrontation. During this exposure, all subject mice were defeated and showed signs of subordination (i.e., lying on their backs, freezing, or showing upright submissive postures). The social defeat procedure lasted for 10 consecutive days. We checked the wounds every time after social defeat bout. The mice with wound size greater than 1 cm were supposed to be removed based on the recommendation by previously study [27]. One mouse was dead and two wounded mice were treated with betadine and excluded from the experiments. After the 5 min defeat session, the experimental mice were placed on the one side of the divider for 24 h with sensory but not physical contact. This procedure was repeated by using a different aggressor CD1 mouse each day for 10 consecutive days. Control mice were pair-housed in equivalent cages separated by a Plexiglas divider and never contacted to CD1 mice. The pairing mouse was different each day. 

#### 2.3.2. Social Avoidance Test

The defeated mouse was placed in interaction box (42 × 42 cm) with an empty wire mesh cage (10 × 4.5 cm) located at the one end. The first session was performed without CD1 mouse in the wire mesh cage. Movement of the defeated animal was tracked for 2.5 min. After 1 min interval, a novel CD1 mouse was introduced into the wire mesh cage and the same defeated animal from first session was placed into the box and tracked for another 2.5 min. The total time spent by the experimental mouse in an 8 cm wide corridor surrounding the wire mesh cage (interaction zone) was automatically measured by SMART software (Panlab, Barcelona, Spain). The interaction ratio was defined as 100 × (interaction time with a target mouse present)/(interaction time without a target mouse present). An interaction ratio of 100 was used as the cut-off value, where scores <100 were defined as “susceptible (Sus)” and scores ≥ 100 as “unsusceptible (Uns)” for both WT and KO mice based on previous studies [25,28].

#### 2.3.3. Open Field Test (OFT)

The open field test (OFT) was used to measure spontaneous locomotor activities such as locomotion time, total distance traveled, and time spent in the central zone (defined as 25% of the total area of the box). Automatic recording and analysis of locomotor activities were achieved in an open acrylic box (30 × 40 × 50 cm) using a video tracking system with SMART software (Panlab, Barcelona, Spain). 

#### 2.3.4. Novel Object Recognition Test (NORT)

Novel objects recognition (NOR) was used to test non-spatial memory in rodents. Initially, mice were habituated (10 min/day) in the empty open chamber (30 × 40 × 50 cm) under the dimly light (20 lux) for 3 days. On day 4, mice were allowed to explore two identical objects placed in the northeast or northwest corner approximately 10 cm from the chamber wall for 10 min during a training trial. One hour later, mice were exposed to testing trial in which they were allowed to explore one familiar object and one new novel object for 10 min. Mice with total exploratory times (TETs) of <5 s per object [29], or showing more than a 30% difference between two objects within 10 min during training trials, were excluded. The recognition index (RI) was the time that animals spent with the novel object (TN) divided by the total time spent exploring the familiar objects (TF) and TN in the testing trial (i.e., RI = TN/(TN + TF)). 

#### 2.3.5. Social Interaction Test 

A C57BL/6J mouse was paired with an unfamiliar CD1 male mouse and their behaviors were recorded for 10 min under dim light (40 lux). Male CD1 mice, aged 4–5 weeks, had a similar weight to experiment mice. The time the experiment mouse spent interacting with the CD1 mouse was manually scored. The following four categories of behaviors were analyzed: neutral (rearing, cage or air sniffing, and self-grooming), social (social grooming/sniffing, nose sniffing, anogenital sniffing, and following/approaching), dominant (attacks, bites, aggressive chasing, climbing, mounting, upright or sideways offensive posture, aggressive grooming and tail rattling) and submissive behaviors (upright or sideways defensive posture, crouching, upright or sideways submissive posture, full submission posture, passive anogenital sniffing or being sniffed at the body part, avoidance, and curling up in the corner and remaining motionless).

### 2.4. Immunohistochemistry (IHC)

After perfusion and fixation, free-floating tissue sections were treated with peroxidase-blocking solution (S2023, Dako, Glostrup, Denmark) and incubated in protein-blocking solution (X0909, Dako). The sections were incubated with a primary antibody against *STMN1* (1:200, ab52630; Abcam, Cambridge, UK) and biotinylated secondary antibodies (1:200, E0466; Dako). After washing with phosphate buffered saline, sections were placed in streptavidin-horseradish peroxidase solution (1:200, P0397; Dako) and incubated with the chromogen AEC (3-amino-9-ethylcarbazole, K3461, Dako). 

### 2.5. Western Blotting 

#### 2.5.1. Preparation of Brain Tissue for Western Blot

After brain extraction, target regions were immediately dissected out on an ice plate according to the Bregma zero coordinates: Amygdala (AMY, −1.05~−2 mm), dorsal striatum (dST, +1.04~−1.46 mm), hippocampus (HIP, −1.5~−2.18 mm) and prefrontal cortex (PFC, +1.54~+1.98 mm) [30] by a 1.0-mm Harris Uni-Core micro-punch (Electron Microscopy Sciences; Hatfield, PA 19440, USA). Bilateral punches tissues were pooled from each mouse. Each tissue sample was quickly cryopreserved in liquid nitrogen and stored at −80 °C until assay. 

#### 2.5.2. Total *STMN1* and *STMN1* Phosphorylated at Serine 16 (pS16-*STMN1*)

After tissue homogenization, protein samples (20 µg) were loaded onto 12% polyacrylamide gels. After transfer, the membranes were treated with 0.25% glutaraldehyde at room temperature for 10 min in 0.2% Tween 20/tris-buffered saline (TTBS) to crosslink tissue proteins that might destroy or mask immunogenic epitopes within the tissue [31,32]. The membranes were then blocked with 5% skimmed milk and incubated with a rabbit monoclonal antibody against *STMN1* (ab52630; Abcam) diluted 1:50,000, and with a rabbit polyclonal antibody phosphorylated at S-16 (3353; Cell Signaling Technology, Danvers, MA, USA) and diluted 1:1000 at 4°C overnight. After washing five times with TTBS, the membranes were incubated with peroxidase-labeled goat anti-rabbit IgG (H + L) (Vector Laboratories, Burlingame, CA, USA) diluted 1:5000 at room temperature for 2 h. 

#### 2.5.3. Short (D2S) and Long (D2L) Forms of The Dopamine D2 Receptor (D2R)

Two isoforms of the D2R, D2L and D2S have been analyzed previously and a few modifications were observed [33,34]. A detailed description of this analysis was reported in our previous study [26]. Glutaraldehyde-treated membranes were incubated in 5% skimmed milk overnight at 4 °C with rabbit polyclonal antibodies against D2L (1:2000) and D2S (1:5000) (AbClon, Inc., Seoul, Korea). After the membranes were washed three times, the primary antibodies were detected using peroxidase-labeled goat anti-rabbit IgG (H + L) (1:3000 for D2L and 1:5000 for D2S; Vector Laboratories) for 2 h at room temperature (25 °C). 

#### 2.5.4. Total Dopamine- and Cyclic Adenosine 3′,5′-Monophosphate-regulated phosphoprotein-32 (DARPP-32), DARPP-32 Phosphorylated at Threonine 34 (pDARPP-32 Thr34), and DARPP-32 Phosphorylated at Threonine 75 (pDARPP-32 Thr75)

Blocked membranes were incubated with the primary antibody, which was rabbit monoclonal total DARPP-32 (1:50,000; Abcam), rabbit monoclonal pDARPP-32 Thr34 (1:1000; Cell Signaling Technology), or rabbit polyclonal pDARPP-32 Thr75 (1:1000; Cell Signaling Technology). After washing three times with TTBS, the primary antibodies were detected using peroxidase-labeled goat anti-rabbit IgG (H + L) antibodies (1:5000; Vector Laboratories) for 2 h at room temperature (25 °C). 

### 2.6. Statistical Analysis

Results were expressed as means ± standard error. The behavioral data were analyzed using two-way analysis of variance (ANOVA) with genotype and group (control, unsusceptible, and susceptible) as the main effects and the change (subtraction of the values after stress from the values before stress) as the dependent variable. If an interaction or main effect was significant, appropriate pairwise comparisons were performed using Tukey’s honest significant difference test. For Western blotting, initial sample sizes were randomly reduced to include 8–15 per group. In addition, the data showing consistent patterns with duplicate were only used for analysis. One-way ANOVA was used to compare groups from each genotype on the Western blots because we could not run all 12 samples (6 groups in duplicate) from two genotypes on a single gel due to difficulties in adjusting the experimental schedule. Fisher’s least-significant-difference post hoc tests were used to validate significant results. In order to confirm *STMN1* gene knock out, two mice from each group of the WT mice and two mice from control group of the KO mice were randomly selected for immunohistochemistry (IHC). Statistical analyses were performed using R software (ver. 3.5.3; R Development Core Team, Vienna, Austria). In all cases, a *p*-value < 0.05 was considered statistically significant. 

## 3. Results

### 3.1. Social Avoidance Test (Table 1)

The proportions of unsusceptible and susceptible mice were not significantly different between the WT and KO mice (*p* = 0.561): 13 (44.83%) unsusceptible and 16 (55.17%) susceptible mice among the WT mice and 12 (37.50%) unsusceptible and 20 (62.50%) susceptible mice among the *STMN1* KO mice. For social interaction ratio, two-way ANOVA revealed significant genotype × group interaction (F_2,79_ = 3.521, *p* = 0.034, post hoc tests: WT-Con vs. WT-Sus, *p* < 0.001; WT-Uns vs. WT-Sus, *p* < 0.001; KO-Con vs. KO-Sus, *p* < 0.001; KO-Uns vs. KO-Sus, *p* < 0.001) and main effect of group (F_2,79_ = 129.737, *p* < 0.001, post hoc tests: Con vs. Sus, *p* < 0.001; Uns vs. Sus, *p* < 0.001) but no significant effect of genotype (F_1,79_ = 0.443, *p* > 0.05). For the corner ratio, two-way ANOVA showed no significant genotype × group interaction and main effect of genotype but a significant main effect of group (F_2,79_ = 17.450, *p* < 0.001, post hoc tests: Con vs. Sus, *p* < 0.001; Uns vs. Sus, *p* < 0.001). 

### 3.2. Open Field Test (Figure 2)

As for the total distance travelled before stress, two-way ANOVA revealed no significant genotype × group interaction (F_2,80_ = 0.879, *p* > 0.05) but significant main effects of genotype (F_1,80_ = 90.747, *p* < 0.001) and group (F_2,80_ = 3.397, *p* = 0.038, post hoc test: Con vs. Uns, *p* = 0.012). After stress, it showed significant genotype × group interaction (F_2,80_ = 3.372, *p* = 0.039, post hoc tests: WT-Uns vs. KO-Uns, *p* < 0.001; WT-Sus vs. KO-Sus, *p* = 0.002) and significant main effect of genotype (F_1,80_ = 36.472, *p* < 0.001) but no significant main effect of group (F_2,80_ = 1.838, *p* > 0.05). For the change, there were no significant genotype × group interaction (F_2,80_ = 1.270, *p* > 0.05) and main effect of genotype (F_1,80_ = 3.180, *p* = 0.078) but a significant main effect of group (F_2,80_ = 4.155, *p* = 0.019, post hoc tests: Con vs. Uns, *p* = 0.039; Con vs. Sus, *p* = 0.025). As for the locomotion time before stress, two-way ANOVA revealed no significant genotype × group interaction (F_2,80_ = 1.741, *p* > 0.05) and main effect of group (F_2,80_ = 1.703, *p* > 0.05) but a significant main effect of genotype (F_1,80_ = 57.076, *p* < 0.001). After stress, it showed significant genotype × group interaction (F_2,80_ = 3.977, *p* = 0.022, post hoc tests: WT-Uns vs. KO-Uns, *p* < 0.001; WT-Sus vs. KO-Sus, *p* < 0.001) and main effect of genotype (F_1,80_ = 39.208, *p* < 0.001) but no significant main effect of group (F_2,80_ = 2.009, *p* > 0.05). For the change, there were no significant genotype × group interactions (F_2,80_ = 2.738, *p* = 0.070) and main effects of genotype (F_1,80_ = 0.126, *p* > 0.05) and group (F_2,80_ = 2.856, *p* = 0.063). As for the time spent in the central zone before stress, two-way ANOVA revealed no significant genotype × group interaction (F_2,79_ = 0.439, *p* > 0.05) and main effect of group (F_2,79_ = 1.410, *p* > 0.05) but a significant main effect of genotype (F_1,79_ = 12.573, *p* < 0.001). After stress, it showed no significant genotype × group interaction (F_2,79_ = 0.837, *p* > 0.05) and main effect of group (F_2,79_ = 1.564, *p* > 0.05) but a significant main effect of genotype (F_1,79_ = 15.682, *p* < 0.001). For the change, there were no significant genotype × group interaction (F_2,79_ = 0.883, *p* > 0.05) and main effects of genotype (F_1,79_ = 0.920, *p* > 0.05) and group (F_2,79_ = 0.377, *p* > 0.05).

In the additional analysis (Supplementary Table S1), baseline locomotor activities (distance traveled and locomotion time) were significantly higher in all three groups of KO mice compared to those of WT mice whereas time spent in central zone was significantly lesser in KO mice compared to WT mice. Comparison of the change between the genotypes showed significant difference only in control group for the distance traveled. 

### 3.3. Novel Object Recognition Test (Figure 3)

As for the recognition index before stress, two-way ANOVA revealed no significant genotype × group interaction (F_2,80_ = 0.613, *p* > 0.05) and main effect of group (F_2,80_ = 0.086, *p* > 0.05) but a significant main effect of genotype (F_1,80_ = 15.263, *p* < 0.001). Genotype difference was also confirmed in the additional analysis (Supplementary Table S2). After stress, it showed no significant genotype × group interaction (F_2,80_ = 1.085, *p* > 0.05) but significant main effects of genotype (F_1,80_ = 14.573, *p* < 0.001) and group (F_2,80_ = 5.114, *p* = 0.008, post hoc tests: Con vs. Uns, *p* = 0.012; Con vs. Sus, *p* = 0.010). For the change, there were no significant genotype × group interaction (F_2,80_ = 0.281, *p* > 0.05) and main effect of genotype (F_1,80_ = 0.213, *p* > 0.05) but a significant main effect of group (F_2,80_ = 3.241, *p* = 0.044, post hoc tests: no significant results). 

### 3.4. Social Interaction Test (Figure 4)

As for the neutral behaviors before stress, two-way ANOVA revealed significant genotype × group interaction (F_2,80_ = 12.998, *p* < 0.001, post hoc tests: WT-Uns vs. KO-Uns, *p* < 0.001; WT-Sus vs. KO-Sus, *p* < 0.001; WT-Con vs. WT-Sus, *p* = 0.032; KO-Con vs. KO-Uns, *p* = 0.002; KO-Con vs. KO-Sus, *p* = 0.005) and main effect of genotype (F_1,80_ = 63.272, *p* < 0.001) but no significant main effect of group (F_2,80_ = 0.729, *p* > 0.05). After stress, it showed no significant genotype × group interaction (F_2,80_ = 1.591, *p* > 0.05) but significant main effects of genotype (F_1,80_ = 19.089, *p* < 0.001) and group (F_2,80_ = 11.385, *p* < 0.001, post hoc tests: Con vs. Uns, *p* = 0.007; Con vs. Sus, *p* < 0.001). For the change, there were significant genotype × group interaction (F_2,80_ = 9.183, *p* < 0.001, post hoc tests: WT-Uns vs. KO-Uns, *p* < 0.001; WT-Sus vs. KO-Sus, *p* < 0.001; WT-Con vs. WT-Sus, *p* < 0.001) and main effects of genotype (F_1,80_ = 86.876, *p* < 0.001) and group (F_2,80_ = 3.223, *p* = 0.045, post hoc tests: Con vs. Sus, *p* = 0.013). Comparison of the change of neutral behaviors between the genotypes revealed significant differences in the Uns and Sus groups (Supplementary Table S3). 

For the social investigation behaviors before stress, two-way ANOVA revealed no significant genotype × group interaction (F_2,80_ = 0.269, *p* > 0.05) and main effect of group (F_2,80_ = 0.052, *p* > 0.05) but a significant main effect of genotype (F_1,80_ = 12.912, *p* < 0.001). After stress, it showed no significant genotype × group interaction (F_2,80_ = 1.424, *p* > 0.05) and main effect of genotype (F_1,80_ = 0.086, *p* > 0.05) but a significant main effect of group (F_2,80_ = 7.197, *p* < 0.01, post hoc tests: Con vs. Uns, *p* = 0.038; Con vs. Sus, *p* < 0.001). For the change, there was no significant genotype × group interaction (F_2,80_ = 1.263, *p* > 0.05) but significant main effects of genotype (F_1,80_ = 10.817, *p* = 0.001) and group (F_1,80_ = 4.123, *p* = 0.019, post hoc test: Con vs. Sus, *p* < 0.013).

For the dominant behaviors before stress, two-way ANOVA revealed no significant genotype × group interaction (F_2,78_ = 0.393, *p* > 0.05) and main effect of group (F_2,78_ = 2.418, *p* = 0.095) but a significant main effect of genotype (F_1,78_ = 5.673, *p* = 0.019). After stress, it showed no significant genotype × group interaction (F_2,78_ = 1.071, *p* > 0.05) and main effects of genotype (F_1,78_ = 2.166, *p* > 0.05) and group (F_2,78_ = 0.940, *p* > 0.05). For the change, there was no significant genotype × group interaction (F_2,78_ = 2.016, *p* > 0.05) but significant main effects of genotype (F_1,78_ = 5.221, *p* = 0.025) and group (F_2,78_ = 4.352, *p* = 0.016, post hoc test: Con vs. Uns, *p* = 0.012). Comparison of the changes of social investigating and dominant behaviors between the genotypes revealed significant differences only in the Sus group (Supplementary Table S3).

For the submissive behaviors before stress, two-way ANOVA revealed no significant genotype × group interaction (F_2,79_ = 2.022, *p* > 0.05) and main effects of genotype (F_1,79_ = 0.952, *p* > 0.05) and group (F_2,79_ = 0.056, *p* > 0.05). After stress, it showed no significant genotype × group interaction (F_2,79_ = 0.150, *p* > 0.05) but significant main effects of genotype (F_1,79_ = 7.506, *p* = 0.007) and group (F_2,79_ = 4.917, *p* = 0.009, post hoc tests: Con vs. Uns, *p* = 0.007). For the change, there were no significant genotype × group interaction (F_2,79_ = 0.153, *p* > 0.05) and main effect of genotype (F_1,79_ = 2.279, *p* > 0.05) but a significant main effect of group (F_2,79_ = 3.402, *p* = 0.04, post hoc tests: no significant results). 

### 3.5. Immunohistochemistry (Figure 5C)

*STMN1* immunoreactivity was strong in the PFC, HIP, and AMY, but weak in the dST, of the WT mice. No *STMN1* immunoreactivity neurons were found in the *STMN1* KO mice.

### 3.6. Western Blotting 

**Figure 5:**
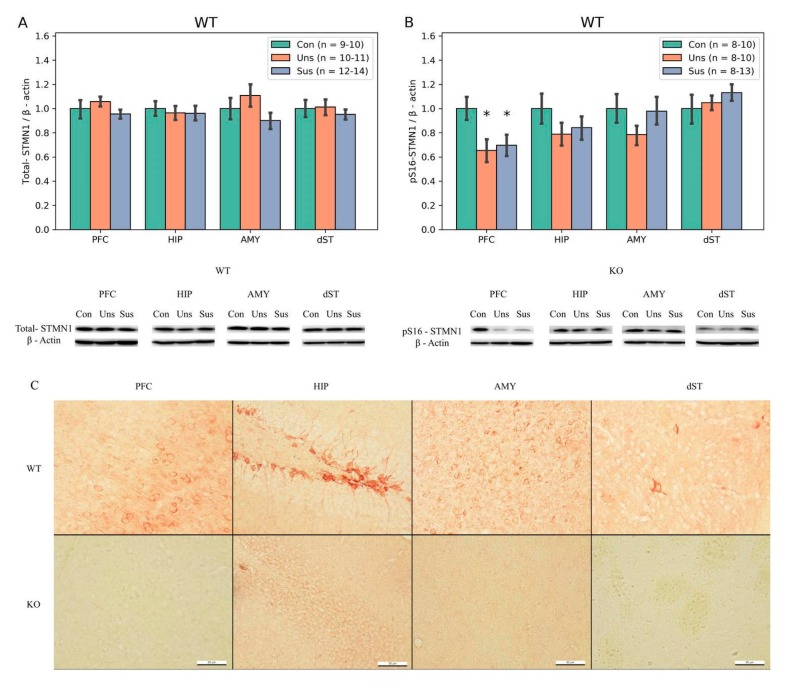
Western blot and immunohistochemistry (IHC) results of stathmin 1 (*STMN1*) among three groups of *STMN1* WT mice. (**A**) Total—*STMN1* and (**B**) pS16—*STMN1*. (**C**) Representative Immunohistochemistry staining images of anti-stathmin 1 (red) in the prefrontal cortex (PFC), dorsal hippocampus (HIP), amygdala (AMY) and dorsal striatum (dST) of WT control. No signal was detected in the KO control in all regions. Scale bar, 50 µm. * *p* < 0.05 for main effect of genotype; Con, Control; KO, Knock Out; Sus, Susceptible; Uns, Unsusceptible; WT, Wild-Type.

Expression of *STMN1* protein was absent in the *STMN1* KO mice (data not shown). There were no significant differences of total *STMN1* expression levels in PFC, HIP, AMY, and dST among the control, unsusceptible, and susceptible WT mice groups. However, significant difference of pS16-*STMN1* levels among the three groups was found in the PFC (F_2,30_ = 3.632, *p* = 0.038). The *post-hoc* tests showed a significance toward lower expression levels in the unsusceptible (*p* = 0.02) and susceptible (*p* = 0.03) groups compared to control group (Figure 5B).

**Figure 6:**
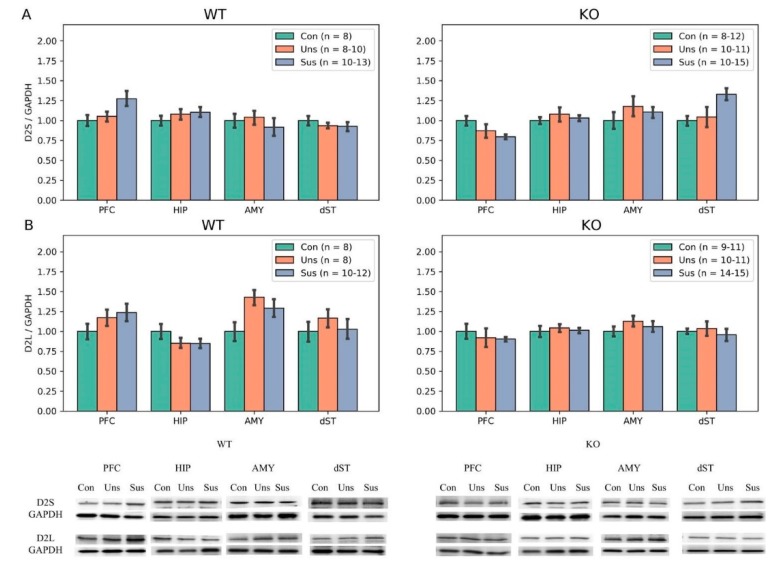
Western blot results of dopamine isoform among three groups in *STMN* WT and KO mice. (**A**) D2S: expression levels of total D2S in the PFC, HIP, AMY and dST; and (**B**) D2L: expression levels of total D2L in the PFC, HIP, AMY and dST. Con, Control; KO, Knock Out; Sus, Susceptible; Uns, Unsusceptible; WT, Wild-Type.

**Figure 7:**
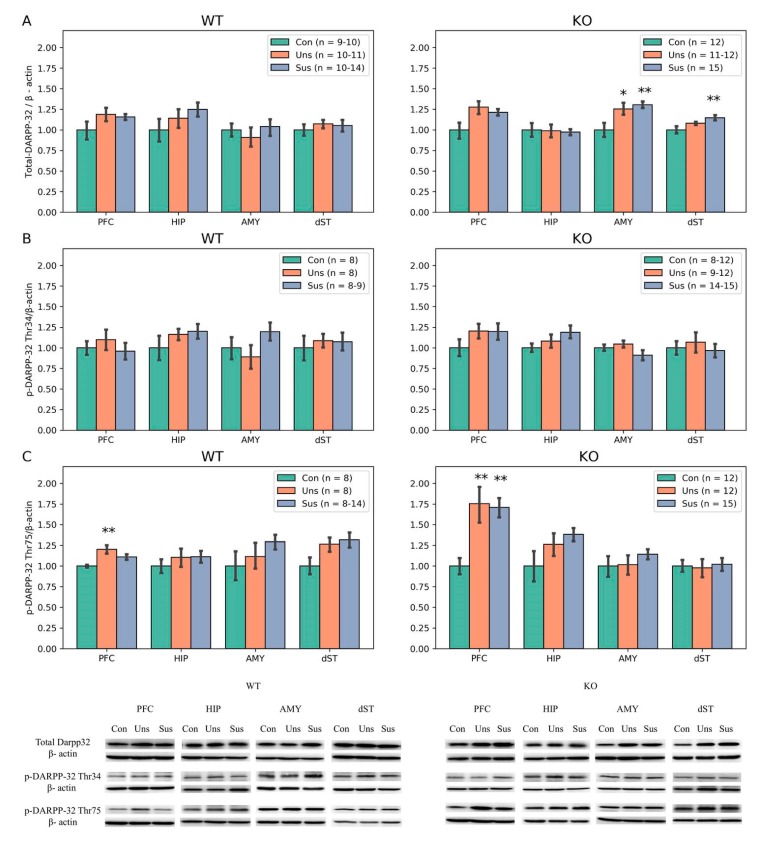
Western blot results of DARPP-32 among three groups in *STMN* KO and WT mice. (**A**) Total DARPP-32: expression levels of total DARPP-32 in the PFC, HIP, AMY and dST of WT and KO mice; (**B**) pDARPP-32 Thr34: expression levels of pDARPP-32 Thr34 in the PFC, HIP, AMY and dST of WT and KO mice; and (**C**) pDARPP-32 Thr75: expression levels of pDARPP-32 Thr75 in the PFC, HIP, AMY and dST of WT and KO mice. * *p* < 0.05, ** *p* < 0.01 compared to control group; Con, Control; KO, Knock Out; Sus, Susceptible; Uns, Unsusceptible; WT, Wild-Type.

Regarding D2S and D2L, there were trends toward altered levels of D2S in the PFC (F_2,23_ = 3.358, *p* = 0.052) and D2L in the AMY (F_2,26_ = 3.207, *p* = 0.056) among the three WT groups. In the *STMN1* KO mice, there were trends toward altered levels of D2S in the PFC (F_2,35_= 3.005, *p* = 0.062) and dST (F_2,25_ = 3.077, *p* = 0.063) among the three groups (Figure 6). For the DARPP-32 expressions in WT mice, significant difference among the three groups was shown only for pDARPP-32 Thr75 in the PFC (F_2,21_ = 6.841, *p* = 0.005). The post hoc tests revealed significantly higher expression in the unsusceptible group than in the control group (*p* = 0.001). In the *STMN1* KO mice, there were significant differences among the three groups for total DARPP-32 in the AMY (F_2,36_ = 5.387, *p* = 0.008) and dST (F_2,35_ = 4.723, *p* = 0.015). The post hoc tests for total DARPP-32 showed significantly higher expressions in the AMY of the unsusceptible (*p* = 0.018) and susceptible (*p* = 0.003) groups compared to the control group, and in the dST of the susceptible group compared to the control group (*p* = 0.004). For pDARPP-32 Thr75 expression in KO mice, significant difference was only found in the PFC (F_2,36_ = 6.943, *p* = 0.002). The post hoc tests showed significantly higher expressions in the unsusceptible (*p* = 0.002) and susceptible (*p* = 0.002) groups compared to the control group (Figure 7).

## 4. Discussion

The *STMN1* gene, known as a fear gene, is an MT-destabilizing phosphoprotein. It is implicated in neurite growth, synaptic plasticity, and the pathophysiology of various neuropsychiatric disorders. We investigated the impacts of *STMN1* KO on behavioral parameters and dopaminergic markers using a mouse model of SDS. Our findings suggest that *STMN1* KO mice have unique behavioral characteristics (e.g., locomotor activities, capacity for recognition, and social interactions) at baseline and show different responses to SDS, in terms of social interactions and dopaminergic markers, compared to WT mice.

### 4.1. Effects of Stathmin 1 Gene Knockout on Behaviors

Before stress, we found significant a main effect of genotype for locomotor activities, NORT and social interaction behaviors (neutral, social investigation and dominant behaviors). Firstly, the results with OFT suggest that *STMN1* KO mice display higher levels of distance traveled and locomotion time and less time spent in the central zone. In the OFT, an animal’s tendency to avoid the center of an arena is considered anxiety-related because the arena center is more threatening than its periphery, and because the administration of anxiolytic drugs increases the likelihood that an animal will occupy the center of the arena [35]. Therefore, these results suggest that *STMN1* KO mice show hyperactivity but are also affected by anxiety. Secondly, the results with NORT indicate that *STMN1* KO mice have lower RIs than *STMN1* WT mice. This suggests the presence of cognitive impairment in *STMN1* KO mice, which contrasts with reports that *STMN*-deficient mice showed normal HIP-dependent spatial memory in a water maze [8]. It should be noted that the Morris water maze test involves a stressful stimulus to ensure that animals are motivated to perform the task, whereas the NORT is based on the natural tendency of mice to spend more time exploring an unknown object than a familiar one [36]. *STMN1* KO mice tend to show subtle cognitive impairments that are only detectable using spontaneous behavior-driven tests. Thirdly, the results with SIT suggest that *STMN1* KO mice display significantly reduced neutral and social investigating behaviors, and increased dominant behaviors compared to WT mice. Rearing is regarded as a useful marker for environmental novelty [37] and self-grooming is a sensitive marker for anxiety and stress levels [38]. Social investigating behaviors are also a measure of social anxiety and preference of engagement in social behaviors. Therefore, these findings may indicate that *STMN1* KO mice do not feel the need to explore their environment or are less vigilant, and experience significant social stress. Taken together, the results with behavioral tests suggest that *STMN1* KO mice show anxious hyperactive behaviors and deficiencies in cognitive function and social interactions. These results contrast with the observation that *STMN1* KO mice develop normally and show no obvious disorders [39].

Regarding the changes induced by SDS, we observed a significant main effect of the group for all behavioral tests, but a significant main effect of genotype was only found for SIT (neutral, social investigating and dominant behaviors). These results suggest that after SDS, effect of *STMN1* KO remains specific to social interacting behaviors, not to locomotion and memory. More specifically, *STMN1* WT mice showed decreased changes of neutral, social investigating and dominant behaviors whereas *STMN1* KO mice demonstrated lesser changes in social interacting and dominant behaviors and even increased change in neutral behavior. These findings may suggest that *STMN1* KO mice are more resistant to the negative effects of SDS on social interaction. The molecular mechanism underlying this resilience to stress may be that *STMN1* deficiency increases MT stability in the AMY, leading to deficiencies in LTP in response to fear induced by defeat stress [8]. 

### 4.2. Effects of Social Defeat Stress on Stathmin Expression

SDS induced no significant difference in total *STMN1* expression among the four brain regions in the control, unsusceptible, and susceptible groups of WT mice. *STMN1* expression in the AMY and HIP reportedly decreased after a single prolonged stress treatment and immobilization stress in rats [40]. In contrast, pS16-*STMN1* expression levels significantly decreased in the PFC of unsusceptible and susceptible groups of WT mice compared to the control group. Phosphorylation of Ser16 or Ser63 appears to be more critical than phosphorylation of Ser25 and Ser38 for the ability of stathmin to bind to soluble tubulin and to inhibit MT assembly in vitro [41,42]. Based on this observation, we focused our work on pS16-*STMN1* expression. Phosphorylation at either Ser16 or Ser63 strongly reduces or abolishes the ability of stathmin to bind to and sequester soluble tubulin resulting in increased MT stability [43]. Increased MT stability is known to contribute to axonal growth, synaptic plasticity, neuronal differentiation and memory [44]. In contrast, dephosphorylation of stathmin at Ser16 has been reported to restrain microtubule polymerization and result in regression of neuronal dendrites [45,46,47]. Taken together, our findings may be interpreted that decreased pS16-*STMN1* expression induced by SDS may lead to inhibiting MT stabilizing activity and cause impairment of axonal transport and synaptic activity. These mechanisms may be associated with various behavioral and molecular abnormalities reported in defeated mice. 

### 4.3. Effects of Stathmin 1 Gene Knockout on Dopaminergic Markers

**Figure 8:**
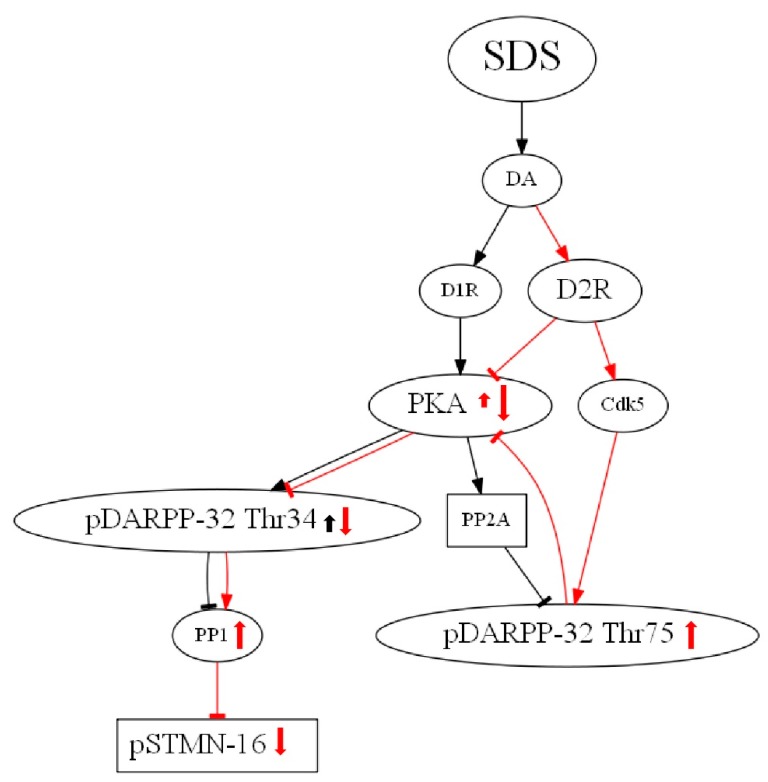
Mechanisms illustrating how social defeat stress may affect expression of *pDARPP-32 Thr34*, *pDARPP-32 Thr75* and *pSTMN-16*.

Following SDS, there was a change in the trends of D2S and D2L expression. In particular, *STMN1* WT and KO mice displayed contrasting changes in D2S expression levels in the PFC. The WT mice in the susceptible group showed increased expression compared to the control group, and *STMN1* KO mice in the susceptible group showed decreased expression compared to the control group. This finding may be related to the different impacts that SDS has on social interactions, especially social investigation behaviors, between WT and KO mice. A significant difference in the expression of total DARPP-32 among the three groups was only observed in the *STMN1* KO mice. Several studies have reported changes in total DARPP-32 expression in rodents after an inhibitory avoidance task [48] and caloric restriction [49]. A functional link has been reported between the microtubule cytoskeleton and dopamine transporter (DAT) [50]. Hence, possible interpretation for the changes of D2S and total DARPP-32 expression in the *STMN1* KO mice may be due to the interaction between stathmin and DAT. Interestingly, the level of pDARPP-32 Thr34 was not changed, but the level of pDARPP-32 Thr75 was significantly increased in the PFC of defeated groups of WT and *STMN1* KO mice. Possible mechanisms may be speculated as follows (Figure 8). Dopamine (DA) released in response to SDS may bind to D1 receptors and increase pDARPP-32 Thr34 via protein kinase A (PKA) activation. At the same time, DA may bind to D2 receptors and increase pDARPP-32 Thr75 via cyclin-dependent kinase 5 (Cdk 5) activation 5 [51]. This also leads to reduction of pDARPP-32 Thr34 via inhibition of PKA. Given that D1 receptor expression was reduced in the frontal cortex of defeated mice [52], a net effect on pDARPP-32 Thr34 would be zero or inhibition. As reduction of pDARPP-32 Thr34 can lead to increased activation of PP-1 [51,53,54] and stathmin at Ser16 is known to be dephosphorylated by PP-1, PP-2A, and PP-2B [55,56], the final effect of SDS would lead to decrease of p*STMN*-16 in the PFC of defeated mice. Understanding how these changes in DARPP-32 may be related to different behavioral profiles at baseline and after defeat stress in *STMN1* WT and KO mice will require further research. 

### 4.4. Limitations

This study had several limitations. First, given that *STMN1* is a cytosolic phosphoprotein, measurement of protein expression using more specific subcellular fraction would have given different results. This should be considered in designing future study. Second, conditioned fear is reported to causes time-dependent biphasic changes in the phosphorylation of *STMN* at Ser16, Ser25, and Ser38 in the dentate gyrus [57]. This finding suggests that phosphorylation levels may change at different time points. Hence, measurement at multiple time points would give more dynamic features. Third, although the behavioral experiment was conducted simultaneously with both genotypes, in the Western blotting experiment we ran the samples on separate gels from both genotypes. Therefore, we were unable to directly compare the Western blotting results between the *STMN1* WT and KO mice. Strength of this study was that we evaluated various behavioral profiles of the *STMN1* KO adult mice extensively before and after stress, together with the unique impacts that SDS had on dopaminergic markers in key regions of the brain implicated in neuropsychiatric disorders. In humans, fear and anxiety as well as cognitive and affective control processing were shown to be associated with *STMN1* polymorphisms [10,14]. An increasing number of correlations are being reported between the *STMN1* gene and a broad range of neuropsychiatric disorders such as schizophrenia [11,12] and depression and attention-deficit/hyperactivity disorder [14]. Exploring the mechanisms of association between *STMN1* KO and altered behavioral and dopaminergic markers would help identify target molecules for the treatment of neuropsychiatric disorders.

## 5. Conclusions

In conclusion, our findings indicate that *STMN1* KO mice display anxious hyperactivity, impaired recognition, and decreased levels of neutral behavior at baseline compared to WT mice. The impacts of SDS on neutral, social investigating and dominant behaviors differed markedly between the *STMN1* WT and KO mice. In addition, different levels of total DARPP-32 and pDARPP-32 Thr75 expression were observed among the control, unsusceptible, and susceptible groups of *STMN1* KO mice.

## Figures and Tables

**Figure 2:**
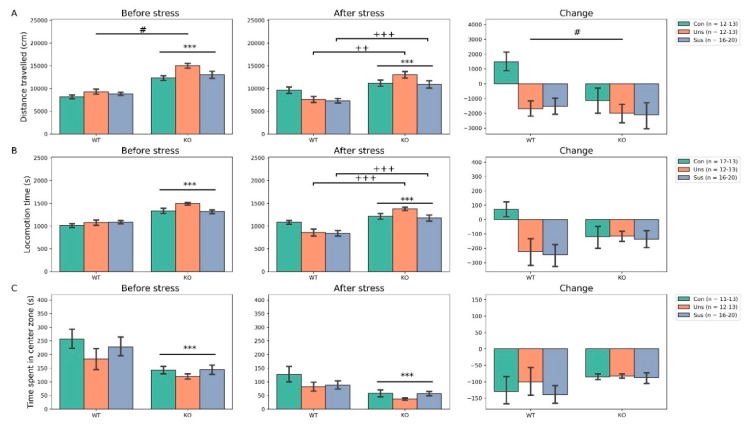
Effects of social defeat on locomotor activities. (**A**) Distance traveled, (**B**) locomotion time, (**C**) time spent in center zone. *** *p* < 0.001 for main effect of genotype; ^#^ *p* < 0.05 for main effect of group; ^++^ *p* < 0.01, ^+++^ *p* < 0.001 for post hoc results of significant interaction; Con, Control; KO, Knock Out; Sus, Susceptible; Uns, Unsusceptible; WT, Wild-Type.

**Figure 3:**
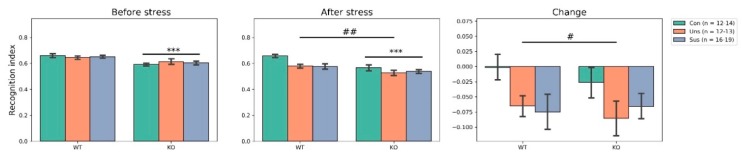
Effect of social defeat on the recognition index (RI) of the novel object recognition test (NORT). *** *p* < 0.001 for main effect of genotype; ^#^ *p* < 0.05, ^##^ *p* < 0.01 for main effect of group; Con, Control; KO, Knock Out; Sus, Susceptible; Uns, Unsusceptible; WT, Wild-Type.

**Figure 4:**
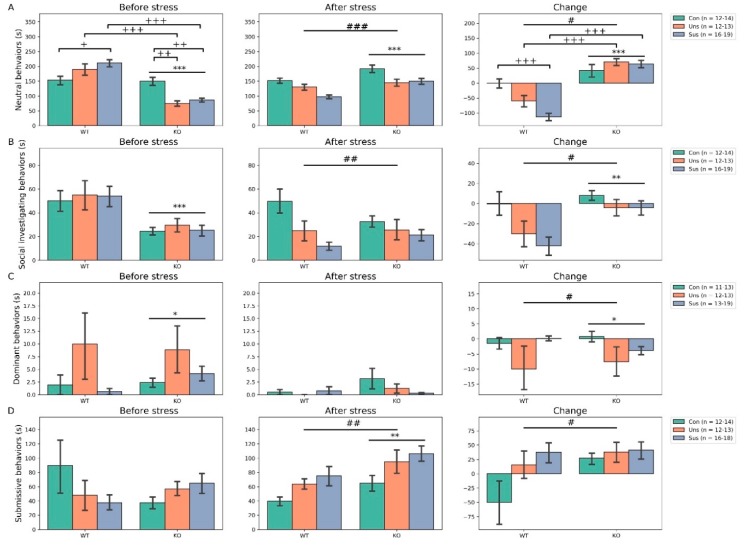
Effects of social defeat on social interaction: (**A**) neutral behavior, (**B**) social investigating behaviors, (**C**) dominant behaviors, (**D**) submissive behaviors. * *p* < 0.05, ** *p* < 0.01, *** *p* < 0.001 for main effect of genotype; ^#^ *p* < 0.05, ^##^ *p* < 0.01, ^###^ *p* < 0.001 for main effect of group; ^+^ *p* < 0.05, ^++^ *p* < 0.01, ^+++^ *p* < 0.001 for post hoc results of significant interaction; Con, Control; KO, Knock Out; Sus, Susceptible; Uns, Unsusceptible; WT, Wild-Type.

**Table 1:** Results of two-way analysis of variance (ANOVA) for social avoidance.

Parameter	Group	WT	KO	*p* ^a^	Group	Genotype	Genotype × Group
Social Interaction ratio	Con	124.247 ± 12.1	146.994 ± 14.6	0.242	F_2,79_ = 129.737, *p* < 0.001	F_1,79_ = 0.443, *p* = 0.507	F_2,79_ = 3.521, *p* = 0.034
	Uns	156.088 ± 13.9	165.356 ± 17.3	0.689			
	Sus	40.986 ± 6.48	22.175 ± 3.19	0.016			
Corner ratio	Con	0.718 ± 0.179	0.675 ± 0.0962	0.836	F_2,79_ = 17.450, *p* < 0.001	F_1,79_ = 0.942, *p* = 0.335	F_2,79_ = 1.599, *p* = 0.209
	Uns	0.938 ± 0.279	1.194 ± 0.334	0.562			
	Sus	5.689 ± 1.13	3.808 ± 0.889	0.204			

Data were expressed in mean ± S.E.M; ^a^ comparison between WT and KO mice by unpaired *t* test. Con, Control; KO, Knock Out; Sus, Susceptible; Uns, Unsusceptible; WT, Wild-Type.

## Supplementary Materials

The following are available online at https://www.mdpi.com/2076-3425/9/9/215/s1, Table S1: Results for locomotor activities, Table S2: Results for NORT, Table S3: Results for Social interaction test, Table S4: Western blot results of Total STMN1, pS16-STMN1/β-actin, Table S5: Western blot results of dopamine D2 receptor isoforms (D2S, D2L), Table S6: Western blot results of total DARPP-32, p-DARPP-32 Thr34, p-DARPP-32 Thr75.

## References

[1] Curmi P.A., Gavet O., Charbaut E., Ozon S., Lachkar-Colmerauer S., Manceau V., Siavoshian S., Maucuer A., Sobel A. (1999). Stathmin and its phosphoprotein family: General properties, biochemical and functional interaction with tubulin. Cell Struct. Funct..

[2] Amat J.A., Fields K.L., Schubart U.K. (1991). Distribution of phosphoprotein p19 in rat brain during ontogeny: Stage-specific expression in neurons and glia. Dev. Brain Res..

[3] Belmont L.D., Mitchison T.J. (1996). Identification of a protein that interacts with tubulin dimers and increases the catastrophe rate of microtubules. Cell.

[4] Felkl M., Leube R. (2008). Interaction assays in yeast and cultured cells confirm known and identify novel partners of the synaptic vesicle protein synaptophysin. Neuroscience.

[5] Hanash S.M., Strahler J.R., Kuick R., Chu E., Nichols D. (1988). Identification of a polypeptide associated with the malignant phenotype in acute leukemia. J. Biol. Chem..

[6] Curmi P.A., Andersen S.S., Lachkar S., Gavet O., Karsenti E., Knossow M., Sobel A. (1997). The stathmin/tubulin interaction in vitro. J. Biol. Chem..

[7] Hayashi K., Pan Y., Shu H., Ohshima T., Kansy J.W., White C.L., Tamminga C.A., Sobel A., Curmi P.A., Mikoshiba K. (2006). Phosphorylation of the tubulin-binding protein, stathmin, by Cdk5 and MAP kinases in the brain. J. Neurochem..

[8] Shumyatsky G.P., Malleret G., Shin R.M., Takizawa S., Tully K., Tsvetkov E., Zakharenko S.S., Joseph J., Vronskaya S., Yin D. (2005). Stathmin, a gene enriched in the amygdala, controls both learned and innate fear. Cell.

[9] Peschanski M., Hirsch E., Dusart I., Doye V., Marty S., Manceau V., Sobel A. (1993). Stathmin: Cellular localization of a major phosphoprotein in the adult rat and human CNS. J. Comp. Neurol..

[10] Brocke B., Lesch K.P., Armbruster D., Moser D.A., Müller A., Strobel A., Kirschbaum C. (2010). Stathmin, a gene regulating neural plasticity, affects fear and anxiety processing in humans. Am. J. Med Genet. Part B Neuropsychiatr. Genet..

[11] Paulson L., Martin P., Persson A., Nilsson C.L., Ljung E., Westman-Brinkmalm A., Eriksson P.S., Blennow K., Davidsson P. (2003). Comparative genome-and proteome analysis of cerebral cortex from MK-801-treated rats. J. Neurosci. Res..

[12] Katayama T., Hattori T., Yamada K., Matsuzaki S., Tohyama M. (2009). Role of the PACAP–PAC1–DISC1 and PACAP–PAC1–stathmin1 systems in schizophrenia and bipolar disorder: Novel treatment mechanisms?. Pharmacogenomics.

[13] Teyssier J.-R., Chauvet-Gelinier J.-C., Ragot S., Bonin B. (2012). Up-regulation of leucocytes genes implicated in telomere dysfunction and cellular senescence correlates with depression and anxiety severity scores. PLoS ONE.

[14] Ehlis A.C., Bauernschmitt K., Dresler T., Hahn T., Herrmann M.J., Röser C., Romanos M., Warnke A., Gerlach M., Lesch K.P. (2011). Influence of a genetic variant of the neuronal growth associated protein Stathmin 1 on cognitive and affective control processes: An event-related potential study. Am. J. Med Genet. Part B Neuropsychiatr. Genet..

[15] Elder G.A., Dorr N.P., De Gasperi R., Gama Sosa M.A., Shaughness M.C., Maudlin-Jeronimo E., Hall A.A., McCarron R.M., Ahlers S.T. (2012). Blast exposure induces post-traumatic stress disorder-related traits in a rat model of mild traumatic brain injury. J. Neurotrauma.

[16] Cao C., Wang L., Wang R., Dong C., Qing Y., Zhang X., Zhang J. (2013). Stathmin genotype is associated with reexperiencing symptoms of posttraumatic stress disorder in Chinese earthquake survivors. Prog. Neuro-Psychopharmacol. Biol. Psychiatry.

[17] Martel G., Hevi C., Wong A., Zushida K., Uchida S., Shumyatsky G.P. (2012). Murine GRPR and stathmin control in opposite directions both cued fear extinction and neural activities of the amygdala and prefrontal cortex. PLoS ONE.

[18] Buwalda B., Kole M.H., Veenema A.H., Huininga M., de Boer S.F., Korte S.M., Koolhaas J.M. (2005). Long-term effects of social stress on brain and behavior: A focus on hippocampal functioning. Neurosci. Biobehav. Rev..

[19] Hollis F., Kabbaj M. (2014). Social defeat as an animal model for depression. ILAR J..

[20] Selten J.-P., van der Ven E., Rutten B.P., Cantor-Graae E. (2013). The social defeat hypothesis of schizophrenia: An update. Schizophr. Bull..

[21] Selten J.-P., Cantor-Graae E. (2005). Social defeat: Risk factor for schizophrenia?. Br. J. Psychiatry.

[22] Lee J.H., Lee S., Kim J.-H. (2017). Amygdala circuits for fear memory: A key role for dopamine regulation. Neuroscientist.

[23] Luo R., Uematsu A., Weitemier A., Aquili L., Koivumaa J., McHugh T.J., Johansen J.P. (2018). A dopaminergic switch for fear to safety transitions. Nat. Commun..

[24] Bagalkot T.R., Jin H.-M., Prabhu V.V., Muna S., Cui Y., Yadav B., Chae H.-J., Chung Y.-C. (2015). Chronic social defeat stress increases dopamine D2 receptor dimerization in the prefrontal cortex of adult mice. Neuroscience.

[25] Jin H.-M., Muna S.S., Bagalkot T., Cui Y., Yadav B., Chung Y.-C. (2015). The effects of social defeat on behavior and dopaminergic markers in mice. Neuroscience.

[26] Prabhu V.V., Nguyen T.B., Cui Y., Oh Y.-E., Lee K.-H., Bagalkot T.R., Chung Y.-C. (2018). Effects of social defeat stress on dopamine D2 receptor isoforms and proteins involved in intracellular trafficking. Behav. Brain Funct..

[27] Golden S.A., Covington H.E., Berton O., Russo S.J. (2011). A standardized protocol for repeated social defeat stress in mice. Nat. Protoc..

[28] Krishnan V., Han M.-H., Graham D.L., Berton O., Renthal W., Russo S.J., LaPlant Q., Graham A., Lutter M., Lagace D.C. (2007). Molecular adaptations underlying susceptibility and resistance to social defeat in brain reward regions. Cell.

[29] McIlwrick S., Pohl T., Chen A., Touma C. (2017). Late-onset cognitive impairments after early-life stress are shaped by inherited differences in stress reactivity. Front. Cell. Neurosci..

[30] Dong H.W. (2008). The Allen Reference Atlas: A Digital Color Brain Atlas of the C57Bl/6J Male Mouse.

[31] Böer U., Buettner F.F., Schridde A., Klingenberg M., Sarikouch S., Haverich A., Wilhelmi M. (2017). Antibody formation towards porcine tissue in patients implanted with crosslinked heart valves is directed to antigenic tissue proteins and αGal epitopes and is reduced in healthy vegetarian subjects. Xenotransplantation.

[32] Gavet O., Ozon S., Manceau V., Lawler S., Curmi P., Sobel A. (1998). The stathmin phosphoprotein family: Intracellular localization and effects on the microtubule network. J. Cell Sci..

[33] Mcdougall S.A., Der-Ghazarian T., Britt C.E., Varela F.A., Crawford C.A. (2011). Postnatal manganese exposure alters the expression of D2L and D2S receptor isoforms: Relationship to PKA activity and Akt levels. Synapse.

[34] Khan Z.U., Mrzljak L., Gutierrez A., De La Calle A., Goldman-Rakic P.S. (1998). Prominence of the dopamine D2 short isoform in dopaminergic pathways. Proc. Natl. Acad. Sci. USA.

[35] Simon P., Dupuis R., Costentin J. (1994). Thigmotaxis as an index of anxiety in mice. Influence of dopaminergic transmissions. Behav. Brain Res..

[36] Ennaceur A., Delacour J. (1988). A new one-trial test for neurobiological studies of memory in rats. 1: Behavioral data. Behav. Brain Res..

[37] Lever C., Burton S., O’Keefe J. (2006). Rearing on hind legs, environmental novelty, and the hippocampal formation. Rev. Neurosci..

[38] Smolinsky A.N., Bergner C.L., LaPorte J.L., Kalueff A.V. (2009). Analysis of grooming behavior and its utility in studying animal stress, anxiety, and depression. Mood and Anxiety Related Phenotypes in Mice.

[39] Schubart U.K., Yu J., Amat J.A., Wang Z.-Q., Hoffmann M.K., Edelmann W. (1996). Normal development of mice lacking metablastin (P19), a phosphoprotein implicated in cell cycle regulation. J. Biol. Chem..

[40] Han F., Jiang J., Ding J., Liu H., Xiao B., Shi Y. (2017). Change of Rin1 and Stathmin in the animal model of traumatic stresses. Front. Behav. Neurosci..

[41] Larsson N., Marklund U., Gradin H.M., Brattsand G., Gullberg M. (1997). Control of microtubule dynamics by oncoprotein 18: Dissection of the regulatory role of multisite phosphorylation during mitosis. Mol. Cell. Biol..

[42] Honnappa S., Jahnke W., Seelig J., Steinmetz M.O. (2006). Control of intrinsically disordered stathmin by multisite phosphorylation. J. Biol. Chem..

[43] Manna T., Thrower D.A., Honnappa S., Steinmetz M.O., Wilson L. (2009). Regulation of microtubule dynamic instability in vitro by differentially phosphorylated stathmin. J. Biol. Chem..

[44] Dent E.W. (2017). Of microtubules and memory: Implications for microtubule dynamics in dendrites and spines. Mol. Biol. Cell.

[45] Ohkawa N., Fujitani K., Tokunaga E., Furuya S., Inokuchi K. (2007). The microtubule destabilizer stathmin mediates the development of dendritic arbors in neuronal cells. J. Cell Sci..

[46] Ohkawa N., Hashimoto K., Hino T., Migishima R., Yokoyama M., Kano M., Inokuchi K. (2007). Motor discoordination of transgenic mice overexpressing a microtubule destabilizer, stathmin, specifically in Purkinje cells. Neurosci. Res..

[47] Wang Y., Wang Y., Dong J., Wei W., Song B., Min H., Teng W., Chen J. (2014). Developmental hypothyroxinaemia and hypothyroidism limit dendritic growth of cerebellar P urkinje cells in rat offspring: Involvement of microtubule-associated protein 2 (MAP 2) and stathmin. Neuropathol. Appl. Neurobiol..

[48] Rosa D.V., Souza R.P., Souza B.R., Guimarães M.M., Carneiro D.S., Valvassori S.S., Gomez M.V., Quevedo J., Romano-Silva M.A. (2008). DARPP-32 expression in rat brain after an inhibitory avoidance task. Neurochem. Res..

[49] Yamamoto Y., Tanahashi T., Kawai T., Chikahisa S., Katsuura S., Nishida K., Teshima-Kondo S., Sei H., Rokutan K. (2009). Changes in behavior and gene expression induced by caloric restriction in C57BL/6 mice. Physiol. Genom..

[50] Wersinger C., Sidhu A.J.B. (2005). Disruption of the interaction of α-synuclein with microtubules enhances cell surface recruitment of the dopamine transporter. Biochemistry.

[51] Reis H.J., Rosa D.V., Guimarães M.M., Souza B.R., Barros A.G., Pimenta F.J., Souza R.P., Torres K.C., Romano-Silva M.A. (2007). Is DARPP-32 a potential therapeutic target?. Expert Opin. Ther. Targets.

[52] Avgustinovichi D., Alekseyenko O. (2010). [^3^H] SCH 23390 Binding in Various Brain Regions of C57BL/6J Mice with Repeated Experience of Victory or Social Defeat in Agonistic Interactions. Physiol. Res..

[53] Nishi A., Shuto T. (2017). Potential for targeting dopamine/DARPP-32 signaling in neuropsychiatric and neurodegenerative disorders. Expert Opin. Ther. Targets.

[54] Greengard P., Allen P.B., Nairn A.C.J.N. (1999). Beyond the dopamine receptor: The DARPP-32/protein phosphatase-1 cascade. Neuron.

[55] Machado-Neto J.A., Saad S.T.O., Traina F. (2014). Stathmin 1 in normal and malignant hematopoiesis. BMB Rep..

[56] Mistry S.J., Heng-Chun L., Atweh G.F. (1998). Role for protein phosphatases in the cell-cycle-regulated phosphorylation of stathmin. Biochem. J..

[57] Uchida S., Martel G., Pavlowsky A., Takizawa S., Hevi C., Watanabe Y., Kandel E.R., Alarcon J.M., Shumyatsky G.P. (2014). Learning-induced and stathmin-dependent changes in microtubule stability are critical for memory and disrupted in ageing. Nat. Commun..

