# Supplementary material for: Effects of Stathmin 1 Gene Knockout on Behaviors and Dopaminergic Markers in Mice Exposed to Social Defeat Stress
Source: Brain Sci. 2019 Aug 26;9(9):215. doi: 10.3390/brainsci9090215 (PMC6769668; doi:10.3390/brainsci9090215)
Supplement: Supplementary file 1 [file brainsci-09-00215-s001.pdf]

Supplementary

Supplementary Table S1 Results for locomotor activities

Parameter	Group	WT			p^a	KO			p^a	p^b	p^c
		Before	After	Change		Before	After	Change			
Distance traveled (cm)	Con	8163.908 ± 453	9655.283 ± 733	1491.376 ± 416	0.126	12324.229 ± 532	11178.705 ± 674	-1145.524 ± 48.5	0.210	< 0.001	0.023
	Uns	9274.856 ± 551	7583.129 ± 657	-1691.727 ± 518	0.006	15039.248 ± 545	13039.852 ± 784	-1999.397 ± 95.9	0.010	< 0.001	0.713
	Sus	8841.456 ± 377	7323.651 ± 498	-1517.805 ± 598	0.020	13026.641 ± 770	10914.460 ± 806	-2112.182 ± 77.9	0.029	< 0.001	0.583
Locomotion time (s)	Con	1013.09 ± 47.6	1083.75 ± 46.5	70.66± 52.1	0.202	1334.945 ± 60	1215.098 ± 64.4	-119.846 ± 73.7	0.143	< 0.001	0.052
	Uns	1078.538 ± 65.3	856.489 ± 79.1	-222.049 ± 95.9	0.039	1493.883 ± 28.3	1378.627 ± 43.4	-115.257 ± 37	0.009	< 0.001	0.314
	Sus	1086.015 ± 39.8	839.773 ± 60.5	-246.243 ± 77.9	0.006	1316.526 ± 47.2	1178.622 ± 67.2	-137.904 ± 60.6	0.034	< 0.001	0.281
Time spent in central zone (s)	Con	256.731 ± 36.4	127.193 ± 26.6	-129.538 ± 41.4	0.013	143.042 ± 14.6	57.811 ± 12.9	-85.231 ± 8.71	< 0.001	0.015	0.337
	Uns	183.462± 37.9	82.545 ± 16.8	-100.917 ± 42.8	0.036	119.337± 9.52	36.903± 4.51	-82.433± 7.79	< 0.001	0.123	0.678
	Sus	228.065± 34.6	88.185 ± 15.1	-139.88 ± 28.3	< 0.001	144.919 ± 17.6	56.994 ± 7.43	-87.925 ± 17.1	< 0.001	0.043	0.464

Data were expressed in mean ± S.E.M; p^a comparison between before and after by paired t test; p^b comparison of before between WT and KO mice by unpaired t test t test; p^c comparison of change between WT and KO mice by unpaired t test. Con, Control; KO, Knock Out; Sus, Susceptible; Uns, Unsusceptible; WT, Wild-Type.

Supplementary Table S2. Results for NORT

Parameter	Group	WT			p^a	KO			p^a	p^b	p^c
		Before	After	Change		Before	After	Change			
Recognition index	Con	0.66 ± 0.0159	0.658 ± 0.0133	-0.002 ± 0.0222	0.945	0.592 ± 0.0115	0.566 ± 0.0235	-0.026 ± 0.027	0.355	0.002	0.495
	Uns	0.645 ± 0.0133	0.58 ± 0.0164	-0.065 ± 0.0183	0.004	0.613 ± 0.0228	0.528 ± 0.0191	-0.086 ± 0.03	0.015	0.247	0.564
	Sus	0.651 ± 0.0122	0.576 ± 0.021	-0.075 ± 0.0292	0.021	0.604 ± 0.0148	0.538 ± 0.0152	-0.066 ± 0.0215	0.007	0.02	0.803

Data were expressed in mean ± S.E.M; p^a comparison between before and after by paired t test; p^b comparison of before between WT and KO mice by unpaired t test t test; p^c comparison of change between WT and KO mice by unpaired t test. Con, Control; KO, Knock Out; Sus, Susceptible; Uns, Unsusceptible; WT, Wild-Type.

Supplementary Table S3 Results for Social interaction test

Parameter	Group	WT			p^{a}	KO			p^{a}	p^{b}	p^{c}
		Before	After	Change		Before	After	Change			
Neutral behaviors(s)	Con	153.086 ± 14.4	152.552 ± 8.62	-0.534 ± 16	0.974	150.668 ± 14	192.265 ± 13.2	41.597 ± 21.4	0.073	0.905	0.128
	Uns	190.313 ± 20.7	130.081 ± 10.2	-60.232 ± 20.8	0.013	75.205 ± 9.61	145.197 ± 12.1	69.992 ± 12.1	< 0.001	< 0.001	< 0.001
	Sus	211.201 ± 12.4	97.327 ± 6.88	-113.874 ± 12.9	< 0.001	86.337 ± 6.71	149.583 ± 10.3	63.246 ± 12.1	< 0.001	< 0.001	< 0.001
Social investigating behaviors (s)	Con	50.218 ± 9.14	49.773 ± 9.96	-0.445 ± 12	0.971	24.514 ± 3.34	32.431 ± 4.98	7.917 ± 4.96	0.134	0.019	0.53
	Uns	54.955 ± 12.6	24.928 ± 9.43	-30.027 ± 13.7	0.049	29.599 ± 5.9	25.467 ± 9.35	-4.133 ± 9.05	0.656	0.085	0.13
	Sus	54.035 ± 9.15	11.943 ± 3.57	-42.093 ± 9.57	< 0.001	25.333 ± 4.76	21.204 ± 4.47	-4.129 ± 6.88	0.566	< 0.001	0.003
Dominant behaviors(s)	Con	1.942 ± 1.94	0.509 ± 0.509	-1.433 ± 2.05	0.499	2.394 ± 0.955	3.17 ± 2.1	0.776 ± 1.83	0.679	0.836	0.08
	Uns	9.971 ± 7.04	0 ± 0	-9.971 ± 7.04	0.182	8.857 ± 4.76	1.24 ± 0.907	-7.617 ± 4.78	0.139	0.897	0.784
	Sus	0.661 ± 0.596	0.786 ± 0.736	0.173 ± 0.967	0.869	4.157 ± 1.38	0.261 ± 0.184	-3.896 ± 1.33	0.010	0.01	0.023
Submissive behaviors(s)	Con	89.934 ± 41.2	39.771 ± 6.88	-50.163 ± 39.4	0.229	37.676 ± 8.85	64.928 ± 11.4	27.251 ± 10.4	0.021	0.238	0.43
	Uns	48.068 ± 21.8	63.822 ± 7.46	15.753 ± 24.5	0.531	56.766 ± 10.7	95.107 ± 17	38.341 ± 17.5	0.05	0.724	0.46
	Sus	37.621 ± 11	75.322 ± 13.8	37.701 ± 17.7	0.049	64.942 ± 13.6	106.328 ± 10.5	41.387 ± 14.3	0.014	0.03	0.875

Data were expressed in mean ± S.E.M; p^{a} comparison between before and after by paired t test; p^{b} comparison of before between WT and KO mice by unpaired t test t test; p^{c} comparison of change between WT and KO mice by unpaired t test. Con, Control; KO, Knock Out; Sus, Susceptible; Uns, Unsusceptible; WT, Wild-Type.

Supplementary Table S4. Western blot results of Total STMN1, pS16-STMN1/β-actin

Protein	Brain region	WT			p^a
		Con	Uns	Sus	
Total STMN1/β-actin	PFC	1 ± 0.079	1.057 ± 0.043	0.955 ± 0.037	0.393
	HIP	1 ± 0.059	0.962 ± 0.058	0.964 ± 0.057	0.393
	AMY	1 ± 0.092	1.109 ± 0.096	0.85 ± 0.080	0.738
	dST	1 ± 0.078	1.013 ± 0.064	0.951 ± 0.043	0.121
pS16-STMN1/β-actin	PFC	1 ± 0.096	0.653 ± 0.097*	0.698 ± 0.086*	0.038
	HIP	1 ± 0.12	0.788 ± 0.0908	0.843 ± 0.082	0.41
	AMY	1 ± 0.10	0.785 ± 0.06	0.979 ± 0.08	0.469
	dST	1 ± 0.116	1.049 ± 0.059	1.131 ± 0.066	0.564

Data were expressed in mean ± S.E.M; *$p<0.05$ versus control group. Con, Control; KO, Knock Out; Sus, Susceptible; Uns, Unsusceptible; WT, Wild-Type.

Supplementary Table S5. Western blot results of dopamine D2 receptor isoforms (D2S, D2L)

Protein	Region	WT			p^a	KO			p^a
		Con	Uns	Sus		Con	Uns	Sus	
D2S /GAPDH	PFC	1 ± 0.083	1.171 ± 0.089	1.237 ± 0.10	0.052	1 ± 0.084	0.921 ± 0.11	0.905 ± 0.025	0.062
	HIP	1 ± 0.087	0.851 ± 0.057	0.849 ± 0.054	0.542	1 ± 0.064	1.043 ± 0.05	1.014 ± 0.0	0.664
	AMY	1 ± 0.10	1.428 ± 0.083	1.293 ± 0.097	0.695	1 ± 0.058	1.128 ± 0.068	1.06 ± 0.058	0.53
	dST	1 ± 0.11	1.168 ± 0.11	1.028 ± 0.11	0.575	1 ± 0.028	1.034 ± 0.090	0.957 ± 0.070	0.063
D2L/GAPDH	PFC	1 ± 0.059	1.054 ± 0.05	1.273 ± 0.07	0.353	1 ± 0.058	0.873 ± 0.085	0.796 ± 0.027	0.685
	HIP	1 ± 0.05	1.079 ± 0.05	1.105 ± 0.04	0.32	1 ± 0.0	1.08 ± 0.08	1.032 ± 0.03	0.845
	AMY	1 ± 0.07	1.042 ± 0.06	0.916 ± 0.10	0.056	1 ± 0.08	1.176 ± 0.11	1.106 ± 0.06	0.471
	dST	1 ± 0.051	0.924 ± 0.030	0.928 ± 0.046	0.627	1 ± 0.049	1.046 ± 0.12	1.329 ± 0.05	0.789

Data were expressed in mean ± S.E.M. Con, Control; KO, Knock Out; Sus, Susceptible; Uns, Unsusceptible; WT, Wild-Type.

Supplementary Table S6. Western blot results of total DARPP-32, p-DARPP-32 Thr34, p-DARPP-32 Thr75

Protein	Region	WT			p^a	KO			p^a
		Con	Uns	Sus		Con	Uns	Sus	
DARPP-32 TOTAL /β-actin	PFC	1 ± 0.10	1.188 ± 0.07	1.157 ± 0.03	0.229	1 ± 0.091	1.277 ± 0.082	1.213 ± 0.034	0.056
	HIP	1 ± 0.13	1.141 ± 0.09	1.249 ± 0.08	0.317	1 ± 0.08	0.988 ± 0.0	0.974 ± 0.03	0.964
	AMY	1 ± 0.07	0.908 ± 0.10	1.038 ± 0.09	0.681	1 ± 0.08	1.253 ± 0.07*	1.304 ± 0.03**	0.008
	dST	1 ± 0.06	1.073 ± 0.04	1.052 ± 0.05	0.747	1 ± 0.04	1.082 ± 0.01	1.147 ± 0.02**	0.015
p-DARPP-32 Thr34 /β-actin	PFC	1 ± 0.06	1.1 ± 0.09	0.958 ± 0.07	0.632	1 ± 0.08	1.202 ± 0.08	1.198 ± 0.08	0.369
	HIP	1 ± 0.12	1.163 ± 0.06	1.201 ± 0.07	0.413	1 ± 0.04	1.081 ± 0.07	1.188 ± 0.06	0.209
	AMY	1 ± 0.1	0.891 ± 0.12	1.195 ± 0.08	0.303	1 ± 0.03	1.046 ± 0.04	0.912 ± 0.05	0.246
	dST	1 ± 0.12	1.086 ± 0.07	1.073 ± 0.09	0.875	1 ± 0.07	1.068 ± 0.1	0.966 ± 0.07	0.765
p-DARPP-32 Thr75 /β-actin	PFC	1 ± 0.01	1.201 ± 0.04**	1.109 ± 0.02	0.005	1 ± 0.09	1.754 ± 0.22**	1.707 ± 0.10**	0.002
	HIP	1 ± 0.07	1.104 ± 0.08	1.112 ± 0.06	0.645	1 ± 0.17	1.263 ± 0.14	1.383 ± 0.07	0.165
	AMY	1 ± 0.1	1.114 ± 0.12	1.292 ± 0.06	0.376	1 ± 0.12	1.014 ± 0.12	1.143 ± 0.05	0.561
	dST	1 ± 0.08	1.264 ± 0.06	1.315 ± 0.06	0.065	1 ± 0.06	0.978 ± 0.11	1.021 ± 0.07	0.944

Data were expressed in mean ± S.E.M; *p<0.05, **p<0.01, ***p<0.001 versus control group of the same genotype. Con, Control; KO, Knock Out; Sus, Susceptible; Uns, Unsusceptible; WT, Wild-Type.
